# BTK inhibitors are a possible emerging therapeutic target for gastric cancer

**DOI:** 10.1016/j.omton.2025.200935

**Published:** 2025-02-03

**Authors:** José Darío Portillo-Miño, Jhon Jairo Calderon, Arnoldo Riquelme

**Affiliations:** 1 Infectious Diseases and Cancer Research Group (GINFYCA), Clinical Research Center, Fundación Hospital San Pedro, Pasto, Nariño, Colombia; 2 Colombian Research Group on Helicobacter pylori, Bogota, Colombia; 3 Grupo de Inmunobiologia y Biologia Celular, Facultad de Ciencias, Pontificia Universidad Javeriana, Bogota, Colombia; 4 Department of Gastroenterology, Faculty of Medicine, Pontifical Catholic University of Chile, Santiago, Chile; 5 Centro para el Control y Prevencion del Cancer (CECAN), Santiago, Chile

## Main text

We read with great interest the article published by Li et al.,^1^ which provided a comprehensive review of promising targeted therapy in gastric cancer (GC) and fascinating molecules with enormous potential. Despite immunotherapy and novel targets expose in the manuscript, considerably enhanced the GC prognosis, the primary and secondary resistance in cancer is formidable for recurrence and is metastasis related.^2^ Moreover, limited outcomes have been observed in patients with GC, probably explained by a resistance to immunotherapy, enriched myeloid-derived suppressor cells (MDSCs), immunosuppressive tumor microenvironment (TME), tumoral molecular heterogeneity subtypes, and chronic inflammation by *H. pylori* infection.^3^^,^^4^^,^^5^ The complexity and heterogeneity of the immunosuppressive TME are critical challenges. In this sense, recent advances in spatial omics research have been fundamental in the understanding of molecular and cellular TMEs of GC, for overcoming immunotherapy resistance and developing novel targeted therapy.^5^ Therefore, it is imperative to develop new strategies that allow the proposed new regimens to improve outcomes and survival in patients with GC. Consistent with this idea, it is suggested that the Bruton tyrosine kinase (BTK) be proposed as an emerging alternative to targeted therapy due to fundamental mechanisms in other cancer types, such as hematologic neoplasms. Its importance relates to being expressed in B cells, and it has an essential role in B cell malignancies.^6^^,^^7^

**Figure 1:**
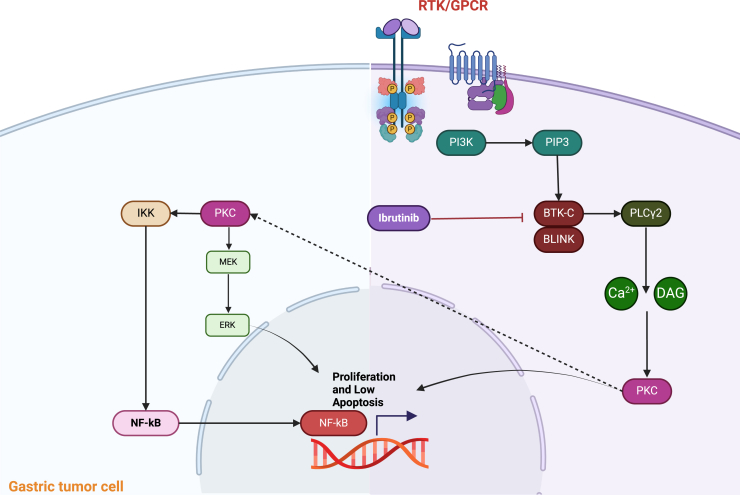
BTK signaling pathways are involved in epithelial tumor cells Source: created with www.biorender.com.

The BTK is a member of the Tec family, a group of cytoplasmic non-receptor tyrosine kinases (BTK, ITK, BMX, and TEC)^8^ and is a soluble tyrosine kinase with essential roles in B cell development, maturation, and signaling.^9^ Moreover, BTK regulates cell proliferation, survival, and migration in B cell tumors. Current studies have demonstrated that BTK is overexpressed in epithelial cancers and plays a crucial pro-tumorigenic role.^8^ Likewise, some research in this field described novel isoforms of the BTK protein expressed in epithelial cancers.^6^ The C-isoform BTK (C-BTK)-encoding transcripts are crucial in the understanding of selectively solid tumors and are expressed in 15% of tumor cells in prostate, bladder, and lung squamous tumor samples.^8^ In this regard, C-BTK is overexpressed in the epithelial tumor cells, activated by the exact molecular mechanism, and signals to the same downstream effectors as A-BTK does in B cells.^6^ Also, A-BTK is overexpressed in solid tumors such as neuroblastoma, glioma, esophageal, gastric, and bladder neoplasms.^6^ Grassilli’s group reported that p65BTK is a truncated isoform that is overexpressed in colon carcinoma cell lines.^8^ Under these circumstances, it has also been discovered that BTK receptors are overexpressed in GC cells. The BTK blockers selectively inhibit the growth of GC cells,^9^ and this is significant because their expression of normal gastric mucosa epithelial cells is low.^9^ BTK-dependent signals from B cell antigen receptors and BTKs can mediate the downstream signaling pathways of G-protein-coupled receptors, antigen receptors, and integrins by functions to cell growth, differentiation, and apoptosis; the BTK induction then triggers the activation of phospholipase C and Ca+ flux, leading to downstream signaling, through transcription factors (nuclear factor κB [NF-κB] and NF-AT) and survival (RAS/Raf, MEK/ERK, and PI3K/AKT) pathways (see Figure 1).^9^ Another BTK-downstream canonical signal pathway is mediated by STAT3 activation, which favors cancer progression. In an ovarian cancer model, BTK has properties for the reduced expression of the JAK2/STAT3 pathway through SOX-2 and BCL-XL.

Recent advances have allowed us to elucidate the key role of the BTK in GC and propose it as an emerging therapeutic target. The BTK inhibitor (LFM-A13) has been shown to be a chemosensitizer and promote apoptosis both *in vitro* and *in vivo* in chemotherapy-resistant B leukemic cells.^6^ On the other hand, Wang et al.^9^ published the first report on the role of BTK and ibrutinib with an application as a target for GC, which proliferation inhibited and phosphorylation decrease of BTK and the signals downstream (PLCγ2, STAT3, and AKT pathways). Additionally, BTK signaling is implicated in survival, apoptosis, cytoskeleton modification, transcriptional regulation, increased glucose uptake, and inflammasome activation through ASC phosphorylation, and this is interesting because BTK can modulate the cancer progression; in this scenario, GC have a immunosuppressive TME and high-inflammatory by *H. pylori* infection,^8^ and BTK has been found to play a crucial role in TME modulation.^10^ For example, in B cells, B cell receptor (BCR) stimulation promotes chemokine receptor type 4 (CXCR4) internalization.^11^^,^^12^ Ibrutinib is anti-BTK that has been approved by the FDA (US Food and Drug Administration) and EMA (European Medicines Agency) for mantle cell lymphoma, marginal zone lymphoma, chronic lymphocytic leukemia, small lymphocytic leukemia, and Waldeström macroglobulinemia.^6^^,^^8^ This is assessed in non-small cell lung cancer (NSCLC) and breast cancer; its functions are related to the capacity to reverse Th2 cell polarization through ITK inhibition and act on cell subpopulations with immunosuppressive properties such as MDSCs, mast cells, and monocytes that are BTK overexpressed. Indeed, it can diminish tumor necrosis factor alpha (TNF-α), interleukin (IL)-1β, and MCP1 (monocyte chemo-attractant protein-1) and decrease peritumoral fibrosis and vascularization.^10^ Ongoing clinical trials for GC are scarce; further research in this field is peremptory for the assessment of ibrutinib and real-world evidence for the established role of this molecule in clinical practice. Other BTK inhibitors (such as acalobrutinib, zanubrutinib, and tirabrutinib) have been approved for hematological malignancies; also, they can represent a good option for epithelial cancers.

The data suggest that BTK inhibitors can be considered as a drug with therapeutic activity in combination with standard therapy, because is involved in multiple mechanism of action that influence TME and canonical and non-canonical signal pathways that regulate of GC biology such as proliferation, angiogenesis, apoptosis, invasion, and immune evasion. However, *in vitro* experiments are still necessary to support carrying out clinical trials and emerging novel therapeutic targets for improving the survival of GC. Therefore, the potential of BTK as a integrated therapeutic target by its capacity to exercise the action on multiple pathways involved in GC is interesting to be explored.

## Declaration of interests

The authors declare no competing interests.
